# Divergence of hydraulic traits among tropical forest trees across topographic and vertical environment gradients in Borneo

**DOI:** 10.1111/nph.18280

**Published:** 2022-06-26

**Authors:** Paulo Roberto de Lima Bittencourt, David C. Bartholomew, Lindsay F. Banin, Mohamed Aminur Faiz Bin Suis, Reuben Nilus, David F. R. P. Burslem, Lucy Rowland

**Affiliations:** ^1^ College of Life and Environmental Sciences University of Exeter Exeter EX4 4QE UK; ^2^ Department of Ecology and Environmental Science Umeå University 90736 Umeå Sweden; ^3^ UK Centre for Ecology & Hydrology Penicuik Midlothian EH26 0QB UK; ^4^ Sabah Forestry Department Forest Research Centre PO Box 1407 Sandakan 90715 Sabah Malaysia; ^5^ School of Biological Sciences University of Aberdeen Aberdeen AB24 3UU UK

**Keywords:** Dipterocarpaceae, hydraulic traits, large trees, niche specialization, Southeast Asia, tree height, tropical forest, water transport

## Abstract

- Fine‐scale topographic–edaphic gradients are common in tropical forests and drive species spatial turnover and marked changes in forest structure and function. We evaluate how hydraulic traits of tropical tree species relate to vertical and horizontal spatial niche specialization along such a gradient.
- Along a topographic–edaphic gradient with uniform climate in Borneo, we measured six key hydraulic traits in 156 individuals of differing heights in 13 species of Dipterocarpaceae. We investigated how hydraulic traits relate to habitat, tree height and their interaction on this gradient.
- Embolism resistance increased in trees on sandy soils but did not vary with tree height. By contrast, water transport capacity increased on sandier soils and with increasing tree height. Habitat and height only interact for hydraulic efficiency, with slope for height changing from positive to negative from the clay‐rich to the sandier soil. Habitat type influenced trait–trait relationships for all traits except wood density.
- Our data reveal that variation in the hydraulic traits of dipterocarps is driven by a combination of topographic–edaphic conditions, tree height and taxonomic identity. Our work indicates that hydraulic traits play a significant role in shaping forest structure across topographic–edaphic and vertical gradients and may contribute to niche specialization among dipterocarp species.

## Introduction

Tropical forests are highly diverse ecosystems hosting an estimated 40 000–50 000 tree species across the biome (Slik *et al*., 2015). Fine‐scale habitat variability is an important driver of tropical forest diversity, structure and function. Within the same microclimatic envelope, differences in edaphic conditions, which are often associated with topographic changes, can lead to almost complete species turnover (Baldeck *et al*., 2013; Schietti *et al*., 2014; Guitet *et al*., 2015). The species turnover is often associated with a sharp change in forest structure and functioning (Lee *et al*., 2002; Cosme *et al*., 2017; Jucker *et al*., 2018). Forest structural changes across fine‐grained topographic–edaphic gradients often follow the same patterns as those found at large spatial scales in the tropics, for example shorter trees in dry sites and on well‐drained and nutrient‐poor soils (Feldpausch *et al*., 2011). This change in forest structure can lead to large differences in biomass across these gradients (Ferry *et al*., 2010; Jucker *et al*., 2018). Topographic–edaphic variability can also drive fine‐scale differences in growth and mortality patterns, drought sensitivity and species reproductive phenology across the landscape (Itoh *et al*., 2003, 2012; Ferry *et al*., 2010; Esteban *et al*., 2021). Due to their fine spatial scale, these gradients are often neglected in large‐scale studies, despite their important influence on tropical forest function. For example, *c*. 40% of forests in Amazonia are situated in valleys and function differently to those in upland plateau environments at the same general location; however, they are treated as a single forest in most vegetation models (Oliveira *et al*., 2019; Esteban *et al*., 2021). As such, understanding the principles governing the evolution, community assembly and function of forests on local environmental gradients is fundamental to understanding and predicting ecosystem processes.

Plant hydraulic traits have significant potential to explain plant species distribution and functioning at both global and local scales (Blackman *et al*., 2011; Trueba *et al*., 2017; Oliveira *et al*., 2019; Laughlin *et al*., 2020). They directly control the efficiency with which plants can transport water and resist soil and/or atmospheric drought, as well as influencing nutrient uptake and carbon assimilation rates (Sperry & Love, 2015; Christoffersen *et al*., 2016). Critically, hydraulic traits constrain the relationship between abiotic (climate and soil) conditions and stomatal conductance (Sperry & Love, 2015; Eller *et al*., 2020). This in turn sets limitations on key aspects of plant architecture such as maximum height, allometry and branching patterns (West *et al*., 1999; Sperry *et al*., 2008; Christoffersen *et al*., 2016) and canopy physiology (Brodribb *et al*., 2017). Hydraulic traits also play a key role in plant evolution and niche specialization (Larter *et al*., 2017; Sanchez‐Martinez *et al*., 2020). In the Central Amazon, rapid species turnover along a fine‐scale topographic gradient (< 1 km) is explained by habitat filtering based on hydraulic traits; trees are highly specialised to the edaphic conditions, with contrasting communities found in drier hills and wetter valleys (Cosme *et al*., 2017; Oliveira *et al*., 2019). Traits related to how efficiently plants transport water and resist drought stress are highlighted as key controls on Amazonian species’ habitat associations (Esquivel‐Muelbert *et al*., 2017; Trueba *et al*., 2017; Oliveira *et al*., 2019); however, whether this is general across the tropics and in the less seasonal Southeast Asian tropical forests is still unknown.

The efficiency with which a plant can transport water through its xylem is often measured as the hydraulic conductivity of the xylem, which is proportional to conduit diameter and length (Sperry *et al*., 2008). Trees located on persistently dry soils are likely to have conservative resource acquisition strategies and are unlikely to need a high degree of hydraulic efficiency for sustaining photosynthesis (Oliveira *et al*., 2021). These trees are usually shorter with narrower and shorter conduits (Santiago *et al*., 2004; Zhang & Cao, 2009; Christoffersen *et al*., 2016; Olson *et al*., 2021). Drier environments are also likely to favour xylem tissue with a greater capacity to resist embolism and therefore hydraulic failure (Choat *et al*., 2007; Blackman *et al*., 2014; Trueba *et al*., 2017; Oliveira *et al*., 2019). Xylem embolism resistance is often measured as the xylem water potential that causes 50% or 88% of water transport capacity loss (P50 and P88, respectively; Cruiziat *et al*., 2002; Pérez‐Harguindeguy *et al*., 2013). Traits that integrate metrics of allocation to water‐demanding and carbon‐acquiring tissues (leaf area) relative to allocation to water supply capacity (xylem area) are also important integrators of these two key traits (Mencuccini *et al*., 2019). Two traits frequently used to quantify this are the leaf : sapwood area ratio (LS), the leaf area allocation per unit transversal area of water transporting tissue, and the leaf specific hydraulic conductivity, which is the xylem hydraulic conductivity per leaf area it supplies. While they are not perfect proxies for hydraulic capacity, as they do not account for branch volume, these traits provide key information on plant hydraulic strategy (Mencuccini *et al*., 2019). All of these hydraulic traits are likely to be strongly affected by soil texture, which is a major determinant of both the amount of soil water available to plants and the hydraulic conductivity connecting water to the roots (Hacke *et al*., 2000; Li *et al*., 2005; Pollacco *et al*., 2020). Sandy soils are likely to be more hydraulically challenging than clay‐rich soils (Hacke *et al*., 2000, p. 200; Li *et al*., 2005; Hultine *et al*., 2006; Poeplau & Kätterer, 2017); however, this may not always be purely related to soil physical properties. The loss of root contact with the soil particles as soils dry can also be a key bottleneck for water uptake from the sandier soils (Herkelrath *et al*., 1977a,b; Li *et al*., 2005; Fisher *et al*., 2007).

Bornean forests have the highest aboveground biomass density of any tropical forest (Avitabile *et al*., 2016), partly because the trees typically attain greater maximum heights and are taller for a given diameter (Banin *et al*., 2012). These features are linked to the prevalence of the Dipterocarpaceae family, which is both highly diverse and contains the tallest known tropical trees on Earth (Brearley *et al*., 2016; Jucker *et al*., 2018; Shenkin *et al*., 2019), attaining maximum heights in excess of 100 m (Shenkin *et al*., 2019). This requires a robust hydraulic system that is capable of overcoming the increase in hydraulic resistance from the soil to the leaves (Sperry *et al*., 2008; Savage *et al*., 2010; McDowell & Allen, 2015). For each 10 m in height, gravity increases the tension in the xylem water column by *c*. 0.1 MPa which, added to the increased resistance from a longer hydraulic pathway, puts taller trees under greater risk of hydraulic failure (Cruiziat *et al*., 2002). Consequently, the hydraulic system of tall trees often differs from that of shorter trees, with taller trees having to develop a more efficient and safer system (Fajardo *et al*., 2019; Liu *et al*., 2019; Olson *et al*., 2021). However, evidence suggests some tall tropical trees either have the same hydraulic traits as shorter trees, or have a less resistant hydraulic system, because of a height related constraint on their capacity to maintain their hydraulic resistance (Rowland *et al*., 2015; Bittencourt *et al*., 2020). If tall trees fail to adjust their hydraulic traits to keep up with the increasing stresses imposed by height, they will rapidly become increasingly susceptible to drought‐induced mortality as they grow (Nepstad *et al*., 2007; Phillips *et al*., 2010; Bennett *et al*., 2015; Ryan, 2015). This could explain why, in Borneo, the tallest dipterocarp species do not occur on soils with a very high sand content, where water is likely more limiting (Herkelrath *et al*., 1977a; Li *et al*., 2005). If true, this group of species may also be most at risk from the drier conditions expected with climate change (Dai, 2013).

Dipterocarps account for a low proportion of Bornean tree diversity (Slik *et al*., 2003) but represent a high proportion of aboveground carbon stocks – up to 60% of forest basal area (Ghazoul, 2016). However, dipterocarps are not found uniformly across the landscape, as the structure of these forests is highly variable across fine spatial scales. The dipterocarp forests in Borneo display marked variation in species composition and function across topographic–edaphic gradients, which are associated with species turnover and changes in forest structure (Paoli *et al*., 2006; Sukri *et al*., 2012; Brearley *et al*., 2016; Jucker *et al*., 2018). In the Sepilok Forest Reserve in Sabah, northeast Borneo, changes in edaphic properties with topography at fine spatial scales (< 1 km), drive transitions from tall forests, growing on nutrient rich and clayey soils, where dipterocarps exceed 80 m in height, to low canopy height forests, located on very sandy soils, where trees reach no more than 40 m in height (Dent *et al*., 2006; Jucker *et al*., 2018). These transitions also lead to an almost complete turnover in dipterocarp species (Nilus, 2004; Brearley *et al*., 2016). Consequently, the species turnover and high beta diversity of these forests can largely be attributed to changing soil conditions. To date, however, there have been limited studies that evaluate how plant traits related to key functional processes, such as water transport and carbon assimilation, vary across such gradients, particularly in Bornean forests. This limits our capacity to understand the physiological and evolutionary drivers of niche specialization. The handful of functional trait studies from topographic–edaphic gradients in Borneo which measure water‐use efficiency, leaf respiration and carbon and nutrient allocation strategies do suggest that there are strong selective pressures on plant function across these gradients, but these have focussed on juvenile size classes of trees and relatively few species (Baltzer *et al*., 2005; Dent & Burslem, 2009, 2016; Russo *et al*., 2010; Katabuchi *et al*., 2012). Tyree *et al*. (1998) present the only study, to our knowledge, of hydraulic traits in Bornean forests. They found no significant change in xylem embolism resistance from more clay rich alluvial to sandier heath forests in Brunei, which supports theoretical expectations that in sites where soil–rhizosphere connections are the hydraulic bottleneck, there is no advantage for trees to have high embolism resistance, unless root : leaf area is simultaneously increased relative to non‐soil–rhizosphere limited sites (Sperry *et al*., 1998). However, Tyree *et al*. (1998) focused only on juvenile trees of both dipterocarp and nondipterocarp species, studying only one individual of 14 distinct species at each site, making it challenging to draw more general conclusions.

**Fig. 1:**
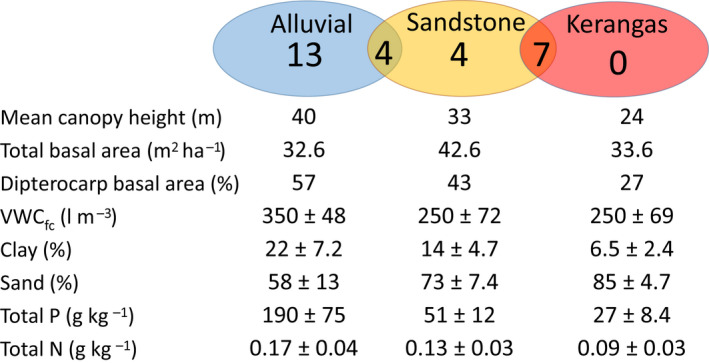
Biotic, physical and chemical descriptions of the three studied forest types (from Nilus, 2004, Jucker *et al*., 2018; Bartholomew *et al*., 2022). The numbers in the Venn diagram indicate the total dipterocarp species richness for species with at least 1 individual ha^−1^ occurring in each forest type. Numbers in the intersections between ellipses indicate the number of species occurring in both environments with at least 1 individual ha^−1^. No species co‐occur in the alluvial and kerangas forests with at least 1 individual ha^−1^. The table shows forest structure indices (mean canopy height, total basal area and percentage dipterocarp basal area in relation to the total for stems ≥ 5 cm diameter at breast height), and soil properties. Forest structure is from a 36 ha forest inventory (12 ha per forest type) with all trees ≥ 5 cm diameter measured; the total number of dipterocarp species in the census is 67, of which 28 have > 1 individual ha^−1^). The description of soil physical and chemical attributes for the different studied forests is presented in terms of mean values ± SD for 75 soil collection points, at 5 cm depth, per forest type. N, nitrogen; P, phosphorus; VWC_fc_, soil volumetric water content at field capacity (VWC measured *c*. 12 h after a strong rainfall event, ensuring enough time for drainage so soil would be saturated and not super saturated).

In this study, we address whether variation in dipterocarp hydraulic traits is determined by topographic–edaphic environments, tree height and their interaction using the edaphic‐topographic gradient in the Sepilok Forest reserve as our study system. We ask whether hydraulic traits controlling embolism resistance (hydraulic safety) and water transport efficiency and capacity can explain the high frequency of niche specialization and spatial turnover of Bornean dipterocarps, as well as the large size differences observed amongst dipterocarp species across habitat types on these gradients. We measured hydraulic traits in 156 individuals of varying heights, starting from 5 m as a minimum threshold, in 13 dipterocarp species across a topographic–edaphic gradient where soils change from clay and nutrient‐rich (alluvial forest) to sandy and nutrient‐poor (sandstone and kerangas forests, sequentially; Fig. 1) across a distance of *c*. 5 km. We tested the following hypotheses:

1 Forests with higher soil water availability (alluvial forests) have selected for dipterocarp species with higher water transport efficiency and lower hydraulic safety (i.e. higher hydraulic conductance, higher LS and lower embolism resistance) than forests on well‐drained sandier soils (sandstone and kerangas forests).
2 Dipterocarp species adjust their hydraulic traits to become safer and more efficient with increasing size, and this adjustment is different across forest types with different soil conditions. We predict that hydraulic efficiency and embolism resistance increase and LSs decrease with tree height, with these traits scaling more strongly in the alluvial forest, where taller stature requires greater hydraulic adjustments.
3 Topographic–edaphic gradients not only select for specific hydraulic traits, but also change the magnitude of hydraulic trait–trait coordination or trade‐offs. We predict the slopes of the relationships between hydraulic traits are steeper in the kerangas forest, as water shortage selects for more extreme values of hydraulic traits in this environment.

## Materials and Methods

### Site and sampling

This study was conducted in the Kabili‐Sepilok Forest Reserve, northeast Borneo (lat. 5°52′34′′N, long. 117°56′58′′E). The landscape is composed of mixed dipterocarp and kerangas forest, with a mean monthly temperature of 26.5 ± 0.6°C (mean ± SD) and a mean annual precipitation of 2640 ± 675 mm (Copernicus Climate Change Service, 2019). Dry spells occur infrequently, although monthly precipitation rarely falls below 100 mm (Supporting Information Fig. S1). Within our site, there are nine permanent 4 ha plots, comprising three plots located in each of the three forest types defined here as alluvial, sandstone and kerangas (Fig. 1; see (Nilus, 2004, for details). The alluvial forest covers the lowland flat valleys and embedded low mudstone hills and is characterised by nutrient rich and clayey soils. The sandstone forest is located on steep hillsides and ridges and is intermediate in terms of soil nutrient and water availability between the alluvial and kerangas forest, with a high diversity of dipterocarp species but lower total dipterocarp canopy height (Jucker *et al*., 2018). The kerangas forest occurs on podzols with associated cuesta dip slopes; soils are nutrient‐poor with a high sand content, and plant communities have a low diversity (see Fox, 1975; Baltzer *et al*., 2005; Dent *et al*., 2006 for details; Fig. 1).

We collected samples from the canopies of 6–16 individuals of different heights from between 4 and 7 dipterocarp species in each forest type (total of 13 species and 156 individuals across all habitats) from July to September 2018. Sunlit branches 1.5–2 m long were cut from the canopy by experienced climbers and transported in moist plastic bags to a laboratory 30–60 min walk from each plot, where they were processed within 1–4 h of being cut to avoid artefacts. Our species selection comprised the most abundant dipterocarp species in each forest type: *Cotylelobium melanoxylon* (Hook.f.) Pierre, *Dipterocarpus acutangulus* Vesque, *Dipterocarpus caudiferus* Merr., *Dipterocarpus grandiflorus* (Blanco) Blanco, *Dipterocarpus kunstleri* King, *Hopea beccariana* Burck, *Parashorea tomentella* (Symington) Meijer, *Shorea johorensis* Foxw., *Shorea macroptera* Dyer, *Shorea multiflora* (Burck) Symington, *Shorea smithiana* Symington, *Shorea xanthophylla* Symington and *Vatica micrantha* Slooten. A summary of the sampling design is presented in Table 1 and species’ abundances per forest type are shown in Table S1. *Shorea macroptera*, *S. smithiana*, *S. multiflora* and *H. beccariana* were abundant in more than one habitat type (dominance rank < 30; Tables 1, S1) and we sampled these in both environments. For each tree, we measured diameter at breast height using a metric tape and tree height using the sine method (Larjavaara & Muller‐Landau, 2013) with a laser distance meter (Forestry Pro II; Nikon, Tokyo, Japan). For wood density and LS, we used an extended dataset with *c*. 10–26 individuals of varying height collected for each of the 13 species in each forest type.

**Table 1:** Summary of traits measured for each dipterocarp species in each environment.

Forest	Species	P50	P88	*K*_s_	*K*_sl_	LS	WD	DBH	Height	H range	*n* embolism	*n K*_s_	*n* WD
Alluvial	*Dipterocarpus caudiferus*	−2.2 ± 0.2	−2.6 ± 0.41	0.6 ± 0.33	0.0017 ± 0.0012	0.00046 ± 0.00012	0.6 ± 0.11	32 ± 36	20 ± 15	7.8–58	9	7	16
	*Dipterocarpus kunstleri*	−2.1 ± 0.32	−2.9 ± 0.54	0.55 ± 0.28	0.0014 ± 0.0013	0.00052 ± 0.00021	0.57 ± 0.053	25 ± 17	19 ± 8.8	7.9–42	12	8	17
	*Parashorea tomentella*	−2.6 ± 0.43	−3.4 ± 0.81	0.62 ± 0.4	0.0016 ± 0.0014	0.00047 ± 0.00021	0.45 ± 0.11	33 ± 25	31 ± 20	7.2–66	16	15	27
	*Shorea johorensis*	−2 ± 0.22	−2.6 ± 0.24	0.91 ± 0.24	0.0015 ± 0.0063	0.00061 ± 0.00016	0.42 ± 0.062	97 ± 27	54 ± 8	38–62	4	4	6
	*Shorea macroptera*	−2.3 ± 0.21	−3 ± 0.28	0.4 ± 0.14	0.0057 ± 0.0028	0.00048 ± 0.0002	0.46 ± 0.076	56 ± 27	20 ± 14	7.5–43	6	4	9
	*Shorea smithiana*	−2.2 ± 0.18	−2.7 ± 0.51	0.67 ± 0.24	0.0024 ± 0.0015	0.00052 ± 0.00024	0.45 ± 0.099	60 ± 30	43 ± 18	15–63	4	4	13
	*Shorea xanthophylla*	−3 ± 0.42	−3.9 ± 0.69	0.26 ± 0.11	0.004 ± 0.0022	0.00062 ± 0.00022	0.52 ± 0.074	41 ± 27	21 ± 8.1	9.5–30	11	11	18
Sandstone	*Dipterocarpus acutangulus*	−2.8 ± 0.33	−3.7 ± 0.65	0.51 ± 0.25	0.0011 ± 0.0083	0.00056 ± 0.00025	0.7 ± 0.064	40 ± 33	25 ± 14	6.7–44	8	13	17
	*Dipterocarpus grandiflorus*	−2.8 ± 0.66	−4.2 ± 0.86	0.34 ± 0.21	0.0011 ± 0.0010	0.00049 ± 0.00027	0.53 ± 0.084	31 ± 30	22 ± 12	7.3–41	14	15	24
	*Hopea beccariana*	−3.2 ± 0.12	−4.2 ± 0.5	0.55 ± 0.26	0.0095 ± 0.0046	0.00051 ± 0.00015	0.61 ± 0.051	31 ± 23	22 ± 13	5.1–40	4	2	14
	*Shorea macroptera*	−2.5 ± 0.63	−3.6 ± 0.88	0.41 ± 0.11	0.0066 ± 0.0033	0.00043 ± 0.00016	0.48 ± 0.058	24 ± 19	19 ± 11	4.4–45	10	4	19
	*Shorea multiflora*	−3.3 ± 0.44	−4.4 ± 0.74	0.32 ± 0.17	0.0062 ± 0.0077	0.00064 ± 0.00033	0.62 ± 0.079	38 ± 32	21 ± 12	6.5–49	10	11	16
	*S. smithiana*	−2.4 ± 0.62	−3.4 ± 0.45	0.38 ± 0.15	0.0071 ± 0.0021	0.00051 ± 0.000078	0.4 ± 0.041	31 ± 24	24 ± 14	9.1–53	10	6	21
Kerangas	*Cotylelobium melanoxylon*	−3.3 ± 0.3	−4.4 ± 0.84	0.69 ± 0.31	0.0026 ± 0.0020	0.00039 ± 0.00021	0.69 ± 0.058	32 ± 18	23 ± 10	7.2–42	14	22	34
	*H. beccariana*	−2.4 ± 0.26	−3.5 ± 0.64	0.74 ± 0.15	0.0033 ± 0.0015	0.00031 ± 0.00014	0.62 ± 0.094	22 ± 12	20 ± 7.4	10–30	4	4	10
	*S. multiflora*	−3 ± 0.64	−4.3 ± 1.1	0.39 ± 0.25	0.0011 ± 0.0010	0.0005 ± 0.00023	0.66 ± 0.042	28 ± 21	19 ± 7	5.2–26	13	20	24
	*Vatica micrantha*	−3.6 ± 0.23	−5.4 ± 0.22	0.33 ± 0.11	0.0010 ± 0.0057	0.029 ± 0.011	0.71 ± 0.048	24 ± 9.4	19 ± 5.4	8.6–29	3	6	14

Data presented are mean ± SD, except for ‘H range’, which reports the minimum and maximum values. ‘*n* embolism’, ‘*n K*_s_’ and ‘*n* WD’ are sample sizes for embolism resistance (P50 and P88); hydraulic conductivity and leaf : sapwood area (*K*_s_, *K*_ls_ and LS); and wood density (WD), respectively.

DBH, diameter at breast height (cm); H range, minimum and maximum tree height (m); height, tree height (m); *K*_s_, maximum hydraulic specific conductivity (kg m m^−2^ s^−1^ MPa^−1^); *K*_sleaf_, maximum hydraulic leaf‐specific conductivity (kg m m^−2^ s^−1^ MPa^−1^); LS, leaf : sapwood area ratio (m^2^ m^−2^); P50, water potential causing a 50% reduction in hydraulic conductivity (MPa); P88, water potential causing an 88% reduction in hydraulic conductivity (MPa); WD, wood density (g cm^−3^).

### Embolism resistance

We measured embolism resistance to xylem tension using the pneumatic method to estimate the amount of xylem embolism and the bench dehydration method to induce embolism (Pereira *et al*., 2016). The pneumatic method measures embolism by extracting air from embolized vessels and quantifying the increase in extracted air during sample desiccation (Zhang *et al*., 2018; Yang *et al*., 2021). While the pneumatic method has not been tested across as many ecosystems as other methods, values of embolism resistance obtained using this method are strongly correlated with those made using other methods and are unlikely to be systematically biased (Pereira *et al*., 2016; Zhang *et al*., 2018; Sergent *et al*., 2020; Guan *et al*., 2021; Paligi *et al*., 2021).

We used terminal branches 40–70 cm long for the pneumatic method measurements. We recut collected samples under water and left them to rehydrate overnight before starting measurements. The following day, samples were connected to an automated pneumatic apparatus (M‐Pneumatron) which applies a vacuum and monitors pressure in the sample for 150 s (Pereira *et al*., 2019). We calculated the volume of gas discharged from the sample for each measurement using the ideal gas law. We calculated the percentage gas discharge (PGD), an estimate of xylem embolism and a proxy of xylem percentage loss of hydraulic conductance, by standardizing each gas discharge measurement of a sample by its minimum and maximum value. We used three M‐Pneumatrons, each measuring the gas discharge of 10 samples for 2–4 d, depending on sample desiccation time, with each sample being measured once every 30 min. We stopped measuring a sample when its water potential was < −9 MPa (Bittencourt *et al*., 2018).

We estimated xylem water potential by measuring the water potential of leaves equilibrated with the xylem by stopping their transpiration. To allow for this equilibration period to take place, the branches were bagged in thick plastic sacks for 1 h, before leaf water potential was measured with a pressure chamber (Model 1515D; PMS, Albany, NY, USA; Scholander *et al*., 1964). We used P50 and P88, the water potential values leading to 50% and 88% hydraulic failure in the xylem, as indices of xylem embolism resistance; they were estimated by fitting PGD to xylem water potential using a sigmoidal equation and extracting the estimated P50 and P88 data points (Pammenter & Vander, 1998; Fig. S2).

### Maximum hydraulic conductance

We measured maximum hydraulic conductivity (*K*_s_) on small (3–5 cm long) distal branch samples using the hydraulic setup described by Sperry *et al*. (1988). This method consists of measuring the sample conductance by applying a known water pressure and quantifying the water flow rate it causes through the sample. Sample conductance is then standardized to sample specific conductivity (*K*_s_) by multiplying by its length and dividing by its transverse area. An ideal measurement of *K*_s_ requires all vessels in the sample to be closed, so the conductance is representative of both vessel lumen and pit membrane conductivity. For tropical trees, mean vessel length is often longer than 10 cm and maximum vessel length is often longer than 1 m (Jacobsen *et al*., 2012), which makes measurement of samples with all vessels closed unfeasible. Thus, we decided to use the shortest samples possible (3–5 cm), to ensure that most of the vessels were open and to reduce bias associated to this (Melcher *et al*., 2012). When using very short samples, while species with different vessel lengths will still have some difference in the percentage of closed vessels, most of the vessels will still be open and this difference is unlikely to cause biases. Using the *K*_s_ measured in this way (vessel lumen *K*_s_) should approximate potential maximum conductivity estimated from vessel diameter and number and provides an internally consistent dataset allowing us to identify patterns in the data, with the caveat that the values should not be compared with *K*_s_ measured in samples with closed vessels provided in other studies.

We flushed samples at *c*. 150 kPa for 30 s before conductance measurements to remove any emboli. Immediately after flushing, we measured sample conductance with a pressure of *c*. 150 kPa for another 30 s. Repeated flushing of a subset of samples for 30 s and measuring their conductance again did not lead to a further increase in conductance, indicating the 30 s flush was sufficient for the removal of all emboli. We used a custom thermal water flow meter in series with the sample to measure water flow (Miller & Small, 1982; Ashauer *et al*., 1999; Methods S1).

### Wood density and leaf : sapwood area

We measured wood density in 1–2 cm diameter samples by dividing their dry mass by their fresh volume. We removed the bark, rehydrated the samples overnight and measured their fresh volume by water volume displacement. We then dried the samples at 60°C for 48 h and measured their dry weight using an analytical scale. We measured the LS on the same samples used for *K*_smax_ measurements. We removed all leaves distal to the measured sample and scanned them to determine area using ImageJ (Schneider *et al*., 2012) and the R package leafarea (Katabuchi, 2015).

### Data analysis

We tested whether the more nutrient‐rich forest types (i.e. higher clay, soil water content and nutrient content) selected for species with more efficient and less safe hydraulic systems (hypothesis 1), and whether tree height affected hydraulic traits and whether tree height effects were modulated by forest type (hypothesis 2) using a linear mixed‐effect model with species as a random variable affecting the intercept and/or the slope of the forest type and tree height fixed effects. We first selected the most parsimonious form of the random effect using the Akaike Information Criterion (AIC), with all fixed effects included. We tested for the significance of the random effect by comparing the full model with a model without random effects and assessed the significance of the difference between the models using a log‐likelihood test (Zuur *et al*., 2009). The contribution of each fixed effect in the model was then tested sequentially using a log‐likelihood test against the full model with one fixed effect removed at a time (Zuur *et al*., 2009; Thomas *et al*., 2017). We tested whether hydraulic traits were interrelated and whether these relationships changed between forest types (hypothesis 3) using standard major axis regression, allowing forest type to affect the slope of the trait–trait relationship through an interaction term. If there was no difference in slopes, we then tested for differences in the intercept.

All analyses were performed in the R statistical environment (v.3.6; R Core Team, 2019). We used the package nlme for linear mixed effect models (Pinheiro *et al*., 2014), the mumin package for the calculation of mixed model marginal and conditional pseudo‐*r* ^2^ values (Barton, 2016) and the smatr package for standard major axis regression (Warton *et al*., 2012). We log transformed *K*_sleaf_ and LS data to meet normality of residuals in mixed model analysis.

## Results

### Variation in hydraulic traits across forest types

**Fig. 2:**
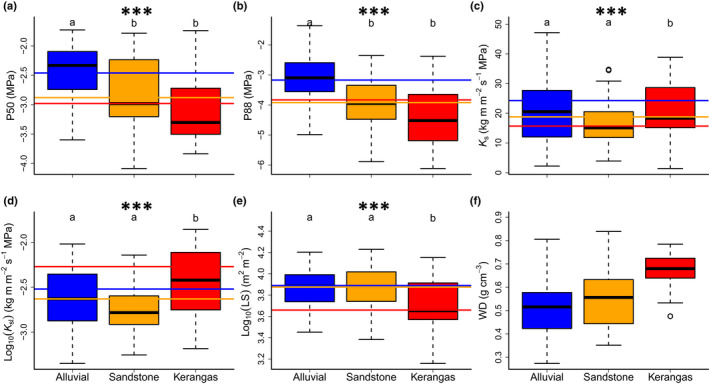
Hydraulic traits of dipterocarp trees measured in three forest types (13 species per forest, *c*. 150 trees samples across all sampled tree heights; significant differences relate to the mixed model analyses, with species as a random effect when significant (see Table 2 for statistics and model structure)). (a, b) Water potential causing 50% and 88% reductions in hydraulic conductivity (P50 and P88); (c) maximum specific hydraulic conductivity (*K*_smax_); (d) maximum leaf specific hydraulic conductivity (*K*_sleaf_); (e) leaf : sapwood area ratio (LS); (f) wood density (WD). The box represents quartiles 1 and 3, with the central line indicating the mean. Whiskers are either maximum values or 1.5× the interquartile range above quartile 3, when outliers are present. Traits for which forest type had a significant fixed effect, within the mixed model analysis, are marked with asterisks (***, *P* < 0.001). Forest types with significantly different trait values are marked with different lowercase letters above their boxes. Note that this figure represents our data distribution along only one of the effects we tested (forest type), while our design and the model we tested are multidimensional (see Table 2). The horizontal lines indicate the mixed model’s group predictions for a 40‐m tall tree for the alluvial, sandstone and kerangas forest types (blue, orange and red lines), when a fixed effect is significant. Different lowercase letters denote significant differences amongst forest type groups in the models. The box represents quartiles 1 and 3, with the central line indicating the median. Whiskers are either maximum values or 1.5× the interquartile range beyond the quartiles, when outliers are present.

Forest type, tree height and their interaction significantly affected hydraulic traits of the dipterocarps we sampled (Fig. 2; Table 2). Embolism resistance varied amongst forest types, such that P50 and P88 decreased progressively from the alluvial (for which mean ± SEM for P50 and P88 were −2.50 ± 0.11 MPa and −3.10 ± 0.15 MPa, Fig. S2) to the sandstone (−2.82 ± 0.14 MPa and −4.3.84 ± 0.19 MPa) and to the kerangas forest (−2.91 ± 0.19 MPa and −4.27 ± 0.28 MPa, *P* < 0.001), following the gradient from periodically wet to consistently dry and freely draining sandy soils underlying these forest types. However, the difference between trees growing in sandstone and kerangas forest was not significant for either P50 or P88 (*P* > 0.10). Trees growing in the kerangas forest had higher hydraulic specific conductivity (*K*_s_), higher leaf specific hydraulic conductivity (*K*_sleaf_) and lower leaf : sapwood area (LS) than those growing in the alluvial and sandstone forests (*P* < 0.001 in all cases). Although median wood density was higher in the kerangas than in other forests (Fig. 2f), wood density was not significantly affected by forest type (*P* = 0.12). We note that pith size variability across wood density samples was negligible in our samples.

**Table 2:** Linear mixed models for six hydraulic traits, explained by the fixed effects of forest type (alluvial, sandstone and kerangas), tree height (m), tree height and forest type interaction (S × Height and K × Height) and random effects accounting for variation in the intercept among species (1¦species) and in the slope of the species‐by‐tree height interaction (species¦height).

Response	Intercept	Height	Fixed effects				*P*	Random effects				
			S × Height	K × Height	Sandstone	Kerangas		(1¦species)	(species¦height)	*P*	$R_{m}^{2}$	$R_{c}^{2}$
P50	−2.46 ± 0.13				−0.42 ± 0.15	−0.42 ± 0.19	0.002	0.55	0.01	< 0.001	0.12	0.61
P88	−3.17 ± 0.17				−0.75 ± 0.19	−0.66 ± 0.27	< 0.001	0.41		< 0.001	0.18	0.41
*K*_s_	13.1 ± 3.0	0.28 ± 0.07	−0.18 ± 0.11	−0.53 ± 0.16	1.69 ± 4.13	12.6 ± 4.82	0.002	4.3		0.002	0.14	0.34
log_10_(*K*_sleaf_)	−2.88 ± 0.07	0.009 ± 0.002			−0.11 ± 0.06	0.25 ± 0.06	< 0.001			0.27	0.27	0
log_10_(LS)	3.89 ± 0.03				−0.014 ± 0.04	−0.23 ± 0.04	< 0.001		0.002	0.006	0.34	0.52
WD	0.57 ± 0.03						0.12	0.13	0.002	< 0.001	0	0.70

Values for fixed effects are fitted parameter ± SE of the mean. S × Height and K × Height are, respectively, the sandstone and kerangas forest effect on the height effect (i.e. their interaction). Values for random effects are the SD of the normal distribution describing the random effect. $R_{c}^{2}$ and $R_{m}^{2}$ are the conditional and marginal model coefficients of determination. Blank cells indicate that the effect is not significant.

*K*_s_, maximum hydraulic specific conductivity (kg m m^−2^ s^−1^ MPa^−1^); *K*_sleaf_, maximum hydraulic leaf‐specific conductivity (kg m m^−2^ s^−1^ MPa^−1^); LS, leaf : sapwood area ratio (m^2^ m^−2^); P50, water potential causing a 50% reduction in hydraulic conductivity (MPa); P88, water potential causing an 88% reduction in hydraulic conductivity (MPa); WD, wood density (g cm^−3^).

### Tree height effects on hydraulic traits and their interaction with forest type

**Fig. 3:**
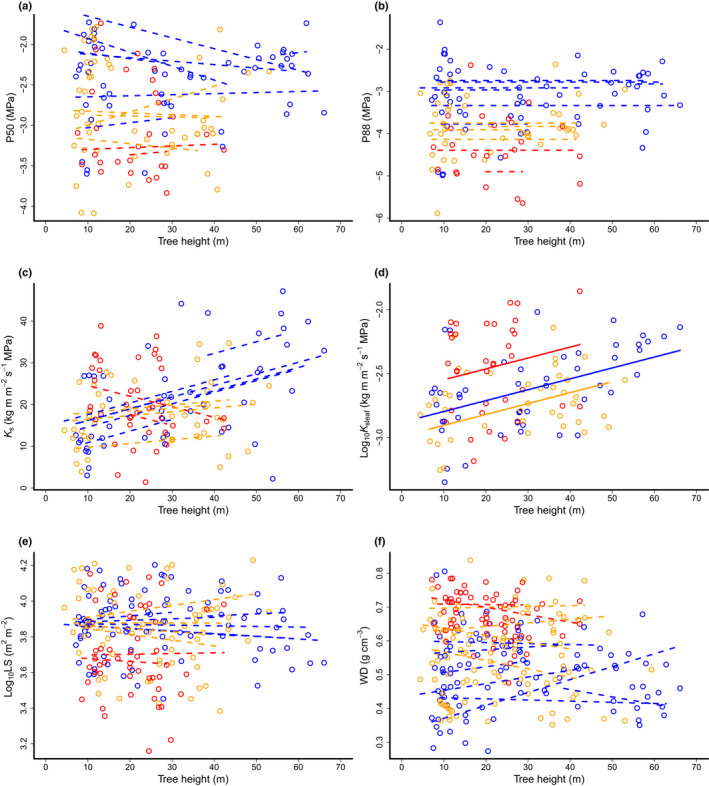
Tree height effects on dipterocarp hydraulic traits. (a, b) Water potential causing 50% and 88% reductions in hydraulic conductivity (P50 and P88); (c) maximum specific hydraulic conductivity (*K*_s_); (d) maximum leaf specific hydraulic conductivity (*K*_sleaf_); (e) leaf : sapwood area ratio (LS); (f) wood density (WD). Data point colour indicates data from different forest types: alluvial (blue), sandstone (orange) or kerangas (red). The significant model effects are represented as lines (see Table 2 for a statistical summary), with dashed coloured lines indicating random plus fixed effects for individual dipterocarp species and solid lines in (d) indicating the fixed effect only, as for this variable there was no significant random effect.

**Fig. 4:**
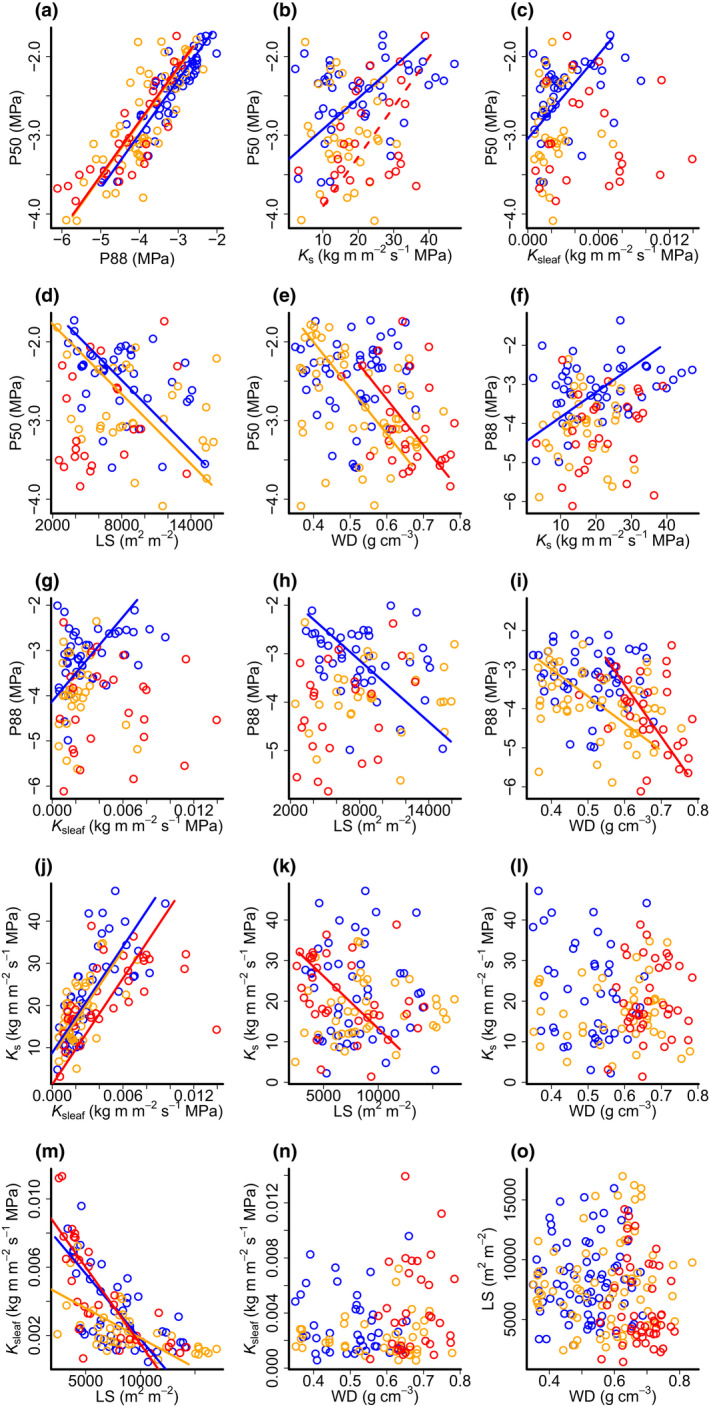
Standardised major axis regression for hydraulic trait interactions and its modulation by forest type. (a–e) Relationship between P50 and P88, *K*_s_, *K*_sleaf_, LS and WD; (f–i) relationship between P88 and *K*_s_, *K*_sleaf_, LS and WD; (j–l) relationship between *K*_s_ and *K*_sleaf_, LS and WD; (m) and (n) relationship between *K*_sleaf_ and LS and WD; (o) relationship between LS and WD. Forest type is represented by data and line colours (alluvial – blue, sandstone – orange, kerangas – red). Continuous coloured lines represent the significant relationships of the fitted models and dashed lines represent marginally significant relationships (0.05 < *P* < 0.06). Significance values and fitted parameters for each model are presented in Table 3. *K*_s_, maximum hydraulic specific conductivity (kg m m^−2^ s^−1^ MPa^−1^); *K*_sleaf_, maximum hydraulic leaf‐specific conductivity (kg m m^−2^ s^−1^ MPa^−1^); LS, leaf : sapwood area ratio (m^2^ m^−2^); P50 and P88, water potential causing 50% and 88% reduction in hydraulic conductivity (MPa); WD, wood density (g cm^−3^).

The heights of the trees we sampled varied from *c*. 5 to 66 m. The slopes of height (*y*) vs diameter (*x*) relationships from standardized major axis regression (SMA) were greater for trees in the alluvial forest than in either the sandstone (*P* = 0.02; Fig. S3) or kerangas (*P* < 0.001) forests, which scaled at a similar rate (*P* = 0.23). Using mixed effects models with tree height, forest type and their interaction as predictors (Table 2; Fig. 3), we found that *K*_s_ and *K*_sleaf_ were dependent on tree height (Table 2; Fig. 3) and that relationships between P50, wood density, LS and tree height varied amongst species (as indicated by the significant random slopes in these models). This suggests that even if we found no global relationship with height across all species, some species are likely to show stronger relationships than others (Fig. 4). There was no effect of height on P88. Maximum hydraulic specific conductivity (*K*_s_) increased by 2.1% per m increase in tree height (*P* < 0.001), and *K*_sleaf_ increased by 1.3% per m increase in tree height (*P* < 0.001; % of *K*_s_ and *K*_sleaf_ in relation to the intercept; for *K*_sleaf_, % is the value back‐transformed from a log_10_ scale). In addition to the direct tree height effect on *K*_s_, with a slope of 0.28 for the intercept (i.e. alluvial forest), there was a significant interaction between forest type and tree height, with the sandstone forest having a shallower slope of 0.10 and the kerangas forest actually exhibiting decreasing *K*_s_ with tree height, with a slope of −0.25 (Fig. 3c).

### Changes in hydraulic trait coordination across forest types

We used SMA to analyse whether the intercept and slope of dipterocarp hydraulic trait relationships were modulated by the forest type in which they occurred. We found that hydraulic trait coordination for many dipterocarp trees varied amongst forest types, either because bivariate trait–trait relationships were only significant in a subset of forest types, or because the relationships had different slopes or intercepts across forests (Fig. 4; Table 3). Among the traits which are expected to be strongly related because they are methodologically linked, *K*_sleaf_–LS had their slope change significantly with forest type (*P* < 0.001; Fig. 4m), while *K*_s_–*K*_sleaf_ and P50–P88 maintained a similar slope across forests, but with varying intercepts (*P* = 0.002 and *P* = 0.021, Fig. 4a,j).

**Table 3:** Results of the standardised major axis regression models, testing whether relationships between dipterocarp hydraulic traits are modulated by forest type (A = alluvial, S = sandstone and K = kerangas).

Hydraulic traits		Coordination‐*P*			Intercept			Slope			Slope‐*P*	Intercept–*P*	Pairwise‐*P*			*r* ^2^		
		A	S	K	A	S	K	A	S	K			A–S	A–K	S–K	A	S	K
P50	P88	**< 0.001**	**< 0.001**	**< 0.001**	−0.23	0.09	0.07	0.69	0.69	0.69	0.2	**0.021**	**0.02**	*0.06*	0.76	0.84	0.55	0.70
P50	*K*_s_	**0.02**	0.36	*0.067*	−3.32		−4.54	0.04		0.06	**0.004**		**0.001**	0.36	**0.04**	0.11		0.11
P50	*K*_sleaf_	**0.002**	0.64	0.55	−3.04			178.2			**< 0.001**		**< 0.001**	0.70	**< 0.001**	0.20		
P50	LS	**0.003**	**0.024**	0.19	−1.31	−1.48		−0.00014	−0.00014		0.33	**< 0.001**	0.15	**< 0.001**	**0.001**	0.19	0.15	
P50	WD	0.86	**< 0.001**	**0.025**		0.33	0.77		−5.83	−5.83	0.30	**0.01**	0.1	0.09	**0.03**		0.19	0.15
P88	*K*_s_	**0.02**	0.41	0.56	−4.47			0.064			**0.014**		**0.011**	0.97	**0.018**	0.10		
P88	*K*_sleaf_	0.01	0.27	0.95	−3.01			2.32			0.33	**< 0.001**	**0.003**	**< 0.001**	**0.003**	0.13		
P88	LS	**0.015**	0.99	0.15	−1.45			−0.0002			**0.034**		0.25	0.08	**0.01**	0.13		
P88	WD	0.42	**0.012**	**0.017**		−0.21	−4.6		−6.93	−13.3	**0.005**		0.46	**0.013**	**0.002**		0.11	0.15
*K*_s_	*K*_sleaf_	**< 0.001**	**< 0.001**	**< 0.001**	96.5	95.1	91.1	12.3	12.3	12.3	0.08	**0.002**	0.47	**< 0.001**	0.04	0.59	0.41	0.43
*K*_s_	LS	0.56	0.54	**0.016**			39.0			0.003	**0.008**		**0.002**	0.08	0.15			0.13
*K*_s_	WD	0.12	0.63	0.58														
*K*_sleaf_ (×10^3^)	LS	**< 0.001**	**< 0.001**	**< 0.001**	9.4	5.3	10.5	0.007	0.003	0.009	**< 0.001**		***< 0.001***	0.29	**< 0.001**	0.37	0.33	0.59
*K*_sleaf_ (×10^3^)	WD	0.33	0.97	0.49														
LS	WD	0.67	0.09	0.11														

Models were fitted with forest type affecting both the intercept and slope of the trait–trait relationships. When trait–trait relationships were significant in at least one forest type (coordination‐*P* < 0.05), we tested for the effect of forest type on the slope of trait coordination. Slope‐*P* is the probability of the trait–trait relationship of the different forest types having the same slope. When forest type did not have a significant effect on the slope of the trait coordination, we refitted the models for the effect of forest type on the intercept of trait relationship. Intercept‐*P* is the probability of the relationship between traits in different forests having the same intercept. Pair‐wise‐*P* is the probability of two different forest types having similar slopes (if slope differences are significant) or intercepts (if intercept differences are significant). *r* ^2^ is the coefficient of determination of the different models. Bold values highlight significant forest‐level interactions, and italics indicate marginally significant interactions (*P* < 0.07). For nonsignificant relationships, entries are left blank. Note that the left hydraulic traits column was fitted as the *y*‐axis in the model.

A, alluvial forest; K, kerangas forest; *K*_s_, maximum hydraulic specific conductivity (kg m m^−2^ s^−1^ MPa^−1^); *K*_sleaf_, maximum hydraulic leaf‐specific conductivity (kg m m^−2^ s^−1^ MPa^−1^); LS, leaf : sapwood area ratio (m^2^ m^−2^); P50, water potential causing a 50% reduction in hydraulic conductivity (MPa); P88, water potential causing an 88% reduction in hydraulic conductivity (MPa); S, sandstone forest; WD, wood density (g cm^−3^).

For the hydraulic traits that are methodologically independent, the P50–*K*_s_ and P88–*K*_s_ coordinated with significant positive slopes, but only for the alluvial forest (*P* = 0.004 and *P* = 0.014; Fig. 4b,f); P50–*K*_sleaf_ and P88–*K*_sleaf_ also had a positive slope in the alluvial forest (*P* < 0.001; Fig. 4c,g). The P50–LS coordination was significant for the alluvial and sandstone forests (*P* = 0.003 and *P* = 0.024; Fig. 4d), with similar slopes and intercepts (*P* = 0.15), indicating that the relationship was consistent across forest types. The P50–WD and P88–WD relationships were significant and negative in the sandstone and kerangas forests, but not in the alluvial forest (Fig. 4e,i), with different intercepts for P50–WD and different slopes for P88–WD (*P* = 0.01 and *P* = 0.005). *K*_s_ decreased with LS for the kerangas forest only (*P* = 0.16; Fig. 4k), while *K*_sleaf_ decreased with LS in all forest types (Fig. 4m), and different forests had different slopes (*P* < 0.001). *K*_s_, *K*_sleaf_ and LS were not related to WD for any forest type (*P* > 0.10; Fig. 4l,n,o).

## Discussion

We investigated how hydraulic traits varied in trees growing in climatically identical but edaphically varied forest habitats in Borneo to identify whether variation in these traits is explained by differential species habitat associations and tree size. Our data reveal that variability in the hydraulic traits of dipterocarp trees is being driven by a complex combination of topographic–edaphic conditions, tree height and taxonomic identity. Embolism resistance and hydraulic efficiency correspond to the edaphic habitat preferences of different dipterocarp species, with plants in drier conditions having higher embolism resistance and lower hydraulic efficiency. This could be limiting the niche space they can occupy across the gradient from wet clay soils to drier, sandy soils. By contrast, we find that only traits related to water transport efficiency and capacity vary with height, most likely because they are key traits enabling taller trees to adjust to greater height‐induced hydraulic limitations. These results suggest that dipterocarp hydraulic traits show independent adaptive responses to both tree height and topographic–edaphic conditions. Furthermore, hydraulic trait–trait relationships were also modulated by forest type, suggesting that selection by topographic–edaphic conditions occurs not only on singular traits, but on multivariate trait relationships which determine plant function. Our data suggest that hydraulic traits play a significant role in shaping the topographic–edaphic and the canopy position dipterocarps species specialize to.

### Embolism resistance explains dipterocarp species niche specialization

Alluvial forest dipterocarp species exhibited the greatest vulnerability to embolism, as measured by P50, with values on average *c*. 0.42 MPa higher than those found in the kerangas and in the sandstone forest dipterocarps. Embolism resistance is known to be strongly selected for by climatological aridity, with drier sites driving the evolution of species with lower P50 (Blackman *et al*., 2014; Larter *et al*., 2017). As the studied sites share the same climate, the large differences in soil conditions along the gradient must be driving these functional changes. The sandier soils of the kerangas and sandstone forests are likely to create more water‐limited conditions, compared with the alluvial forest, despite the same climatological water availability. Unlike most of the Amazon, where seasonality can be intense and interannual variability can lead to periodic drought (Lopes *et al*., 2016), monthly precipitation in Bornean forests rarely drops below 100 mm, and water deficits are likely to be relatively minor or occur sporadically, for example in association with El Niño events (Guan *et al*., 2015; Fig. S1). However, these infrequent climatologically dry spells are likely to result in more intense water shortages in the kerangas and sandstone forests, where soils hold less water. Results from one other study looking at plant hydraulic traits across soil gradients in Bornean forests (Tyree *et al*., 1998) showed a similar trend in embolism resistance (P50) for a sandier forest and a forest on a more clay‐rich soil (*P* = 0.11). Their work was based on a much smaller sampling effort (one individual of 14 species per site) and the trees sampled were juveniles (< 5 cm DBH), which could be the reason for its marginally significant results. In the Central Amazon, trees in sandier soils in valleys, close to the water table, were shown in previous studies (Oliveira *et al*., 2019; Fontes *et al*., 2020) to have lower embolism resistance than trees growing on clayey soils farther from the water table; by contrast, we found trees growing on more clay‐rich soils in Sepilok to have lower embolism resistance. Together, these results suggest that water availability rather than soil texture *per se* is most critical in selecting for embolism resistance. If trees in the kerangas forest were to invest in a higher density of fine roots relative to the trees in other forests (which has been observed, D. F. R. P. Burslem, pers. obs.), they may partially overcome the water transport limitation in the soil, as suggested by the model of Sperry *et al*. (1998). This would make xylem adaptations an effective way to prevent embolism and maintain water transport.

However, we also found *K*_s_ and *K*_sleaf_ to be *c*. 96% and *c*. 78% higher for trees growing in the kerangas forest, relative to the alluvial and sandstone forests (Fig. 2). We expected trees in the alluvial and sandstone forests to have higher hydraulic efficiency and capacity than those in the kerangas forest to support their denser canopies and greater heights (Jucker *et al*., 2018). Trees in the kerangas forest also had lower LS, which further increases leaf water supply capacity and highlights the importance of tree allometry to water transport (Martínez‐Vilalta *et al*., 2009; Petit *et al*., 2018; Mencuccini *et al*., 2019). We note these results are not artefacts of different tree sizes in these forests, as we controlled for tree height in our analyses. Unexpectedly, we found no relationship between *K*_s_ and P50 in the kerangas forest, which would suggest that there is no discernible safety–efficiency trade‐off among trees growing in this environment and allows the kerangas forest trees to have both higher *K*_s_ and lower P50 than those in the other forests (Fig. 4). This result, together with the higher embolism resistance of the kerangas forest trees, strongly suggests that the sandier soils in this forest are more hydraulically challenging, requiring both greater embolism resistance to cope with soil water deficits and higher water transport capacity to avoid significant water potential drops and embolism.

Very few dipterocarp species occur in the kerangas forest (Fig. 1), and its tree flora is generally depauperate compared to that of mixed dipterocarp forest across Southeast Asia (Ashton, 2014). These patterns in tree species richness suggest that the traits required for survival in this hydrologically drier site are uncommon within the dipterocarp lineage and are rare within most other families represented in the Southeast Asian tree flora. Dipterocarp species with low embolism resistance and water transport capacity are likely to be constrained to wetter environments, otherwise frequent vessel embolization would either kill them or lead to a very high cost of replacing embolized xylem tissue (Eller *et al*., 2018). This may also explain why individuals of species generally found only in alluvial or sandstone forests are only found in kerangas forests along the banks of streams (from plot inventory data, not shown).

### Changes in hydraulic efficiency and capacity with height

Hydraulic efficiency, as measured by *K*_s_, is a trait linked to how efficiently plants use their xylem volume for water transport (Cruiziat *et al*., 2002; Sperry *et al*., 2008; Bittencourt *et al*., 2016). Greater *K*_s_ allows a plant to supply the same leaf area using a smaller volume of xylem tissue or over a longer distance with the same amount of tissue investment. We found that dipterocarp trees in the alluvial and sandstone forests adjust to being taller by increasing the hydraulic efficiency (*K*_s_) in their terminal branches, resulting in higher water transport capacity (*K*_sleaf_) in the canopies of taller trees (Fig. 3). This indicates that dipterocarp trees compensate for increasing hydraulic resistance with height by adjusting their anatomy, as predicted by scaling studies (West *et al*., 1999; Sperry *et al*., 2008; Olson *et al*., 2009, 2014). Decreases in LS with height were not found (Fig. 3d) in the alluvial and sandstone forests, which suggests that the increases in *K*_sleaf_ with height were driven by adjustments to *K*_s_, rather than morphological adjustments in LS. This capacity to compensate for increased height through adjusting *K*_s_, without adjusting LS, may also allow trees to have more flexibility to maintain their carbon balance (photosynthetic gain from leaf area and respiratory cost of sapwood) and biomechanical support, all of which change with LS (Poyatos *et al*., 2007; Loehle, 2016; Pratt & Jacobsen, 2016; Lehnebach *et al*., 2018). However, we acknowledge that we were only able to measure sapwood area, rather than fully quantifying the hydraulic pathway by measuring branch length and volume, which is likely to drive the supply–demand dynamics within trees more directly (Anfodillo *et al*., 2013). This could explain why we found no relationship between LS and tree height for the kerangas forest trees. Trees in the kerangas forest had a decrease in *K*_s_ with tree height, but the *K*_sleaf_ still increased with tree height, suggesting that adaptations to allometry, in addition to adaptations in hydraulic efficiency, might play an important role in adaptation to maintain leaf water supply function with increasing tree size. Shorter trees having higher *K*_s_ in these forests may be an adaptation to the limiting water conditions of the sandy soil, meaning rapid uptake is needed before the water infiltrates to lower soil layers. The capacity of dipterocarp trees to adapt *K*_s_ and presumably vessel anatomy to achieve greater height is likely to have a lower net carbon cost than adapting allometric ratios (i.e. adding more water transport tissue or decreasing leaf area). It is therefore possible that the maximal limit on this *K*_s_ adjustment may be key in determining maximum tree height in dipterocarps.

Increasing embolism resistance with tree height was dependent on species identity rather than a universal family‐level strategy, as found in Eastern Amazonian tree species (Bittencourt *et al*., 2020). Increases in embolism resistance with height are hypothesised to be a necessary adjustment to compensate for the following: gravity‐induced decreases in tree water potential with height (Bennett *et al*., 2015; Couvreur *et al*., 2018), water potential drops arising from vessel widening not fully compensating for height‐related increases in resistance (Poyatos *et al*., 2007; Liu *et al*., 2019), and declines in water potential associated with the drier atmospheric conditions found higher up in forest canopies (Kumagai *et al*., 2001; Bennett *et al*., 2015). Our results support the idea that adjustments in embolism resistance do not represent a general strategy for trees to deal with water stress from increasing tree height. Our results therefore suggest that different hydraulic traits are being selected for in dipterocarp trees to adjust to vertical vs horizontal variation in environments, with hydraulic efficiency and embolism resistance additively, but independently, modulating dipterocarp hydraulic function.

### Hydraulic trait networks are modulated by the environment of dipterocarp trees

Hydraulic traits are not independent, and variation in one trait can result in changes in another hydraulic trait or influence traits relating to other functions, such as carbon assimilation or biomechanics (Pratt & Jacobsen, 2016; Olson *et al*., 2018). Similarly, multiple distinct selective pressures, such as selection for being tall and for survival on drier soils, may act simultaneously but in different directions on overlapping sets of traits. This could cause significant spatial variation in trait coordination with varying environmental conditions and forest structural changes. We tested the hypothesis that hydraulic trait relationships are not universal, but rather are modulated by forest type, finding multiple instances where trait coordination changed across habitat types within the dipterocarp species we studied (Fig. 4). We found that key hydraulic trait relationships that are commonly reported in the literature, such as P50–wood density and P50–*K*_sleaf_ (Markesteijn *et al*., 2011; Reich, 2014; Christoffersen *et al*., 2016), were altered in terms of slope, intercept and strength between the three forest types. One of the key hydraulic trait relationships, which is highly relevant to the hydraulic niche that a tree occupies, is the trade‐off between hydraulic safety (embolism resistance – P50) and hydraulic efficiency (hydraulic specific conductivity – *K*_s_; Gleason *et al*., 2016). Our results show that this trade‐off seems to be context dependent and only significant for dipterocarps in the alluvial forests (P50; Fig. 4), possibly because *K*_s_ is under stronger selection in an environment that supports trees expressing greater variation in height. This may, in part, explain why global analyses find weak relationships between hydraulic safety (P50) and hydraulic efficiency (*K*_s_) in comparisons across multiple sites that vary in environmental conditions (Gleason *et al*., 2016).

### Conclusions

Dipterocarp hydraulic traits change with both local topographic–edaphic conditions and tree height. Our study shows that embolism resistance varies substantially amongst dipterocarp species found in forests with different topographic–edaphic conditions, under the same climatic conditions. These differences may determine the capacity for species to survive in environments with differential soil water availability and may contribute to the significant spatial turnover and evidence of niche specialization observed along those fine‐scale environmental gradients. Hydraulic capacity was influenced by both habitat type and tree height, resulting from adjustments in hydraulic efficiency, reflecting patterns of tree allometry. The tallest tropical trees therefore did not adjust their capacity for resisting embolisms with height, but increased their water transport capacity to compensate for the increased resistance to water transport with size. The strong habitat relationship with embolism resistance and size relationship with water transport efficiency meant that a universal hydraulic safety–efficiency trade‐off was not found across these forests, although it was expressed within the wetter alluvial forest. Critically, habitat affiliation of the dipterocarp species also had a considerable impact on hydraulic trait relationships, which varied substantially between habitat types. This evidence demonstrates the importance of plant hydraulic traits for controlling both vertical and spatial niche occupancy within Southeast Asian dipterocarp forests.

## Author contributions

PRdLB, LR, LFB and DFRPB conceived the research ideas and developed the project. PRdLB and DCB collected the data. PRdLB analysed the data and wrote the manuscript. PRdLB, LR, LFB, DFRPB, MAFBS and RN contributed to manuscript preparation.

## Supporting information


**Fig. S1** Time series of precipitation (blue, mm), evapotranspiration (orange, mm) and climatological water deficit (red, mm; calculated as in (Barros *et al*., 2019)) for the Sepilok region from 2003 to 2020.
**Fig. S2** Increase in embolism (PGD, percentage air discharge) with increasing branch xylem water potential (Ψ) of dipterocarp individuals in each forest type: (a) alluvial forest, (b) sandstone forest and (c) kerangas forest.
**Fig. S3** Relationship between tree height and diameter for dipterocarp trees of the studied species in the alluvial forest (blue), sandstone forest (orange) and kerangas forest (red).
**Methods S1** Description of the water flow meter used for maximum hydraulic conductance measurements.
**Table S1** Tree density (individuals ha^−1^), species dominance (% basal area) and dominance rank (by basal area) within all species for each of the studied species in each forest type.
Please note: Wiley Blackwell are not responsible for the content or functionality of any Supporting Information supplied by the authors. Any queries (other than missing material) should be directed to the *New Phytologist* Central Office.

## Data Availability

The data that support the findings of this study are available at https://doi.org/10.5061/dryad.w6m905qrs.
