# Supplementary material for: Divergence of hydraulic traits among tropical forest trees across topographic and vertical environment gradients in Borneo
Source: New Phytol. 2022 Jun 26;235(6):2183–98. doi: 10.1111/nph.18280 (PMC9545514; doi:10.1111/nph.18280)
Supplement: Supplementary file 1 — Fig. S1 Time series of precipitation (blue, mm), evapotranspiration (orange, mm) and climatological water deficit (red, mm; calculated as in (Barros et al., 2019)) for the Sepilok region from 2003 to 2020. Fig. S2 Increase in embolism (PGD, percentage air discharge) with increasing branch xylem water potential (Ψ) of dipterocarp individuals in each forest type: (a) alluvial forest, (b) sandstone forest and (c) kerangas forest. Fig. S3 Relationship between tree height and diameter for dipterocarp trees of the studied species in the alluvial forest (blue), sandstone forest (orange) and kerangas forest (red). Methods S1 Description of the water flow meter used for maximum hydraulic conductance measurements. Table S1 Tree density (individuals ha−1), species dominance (% basal area) and dominance rank (by basal area) within all species for each of the studied species in each forest type. Please note: Wiley Blackwell are not responsible for the content or functionality of any Supporting Information supplied by the authors. Any queries (other than missing material) should be directed to the New Phytologist Central Office. [file NPH-235-2183-s001.pdf]

New Phytologist Supporting Information

Article title: **Divergence of hydraulic traits among tropical forest trees across topographic and vertical environment gradients in Borneo**

Bittencourt, P. R. L., Bartholomew, D. C**,** Banin, L. F, Bin Suis, M. A. F., Nilus, R., Burslem, D. F. R. P., Rowland, L.

Article acceptance date: 23 May 2022

The following Supporting Information is available for this article:

Methods S1. Description of the water flow meter used for maximum hydraulic conductance measurements.

Table S1. Tree density (individuals per hectare), species dominance (% basal area) and dominance rank (by basal area) within all species for each of the studied species in each Forest type.

Figure S1. Time series of precipitation (blue), evapotranspiration (orange) and climatological water deficit (red, also in mm; calculated as in (Barros *et al.*, 2019)) for the Sepilok region from 2003 to 2020.

Fig. S2. Increase in embolism (PGD – percentage air discharge) with increasing branch xylem water potential (Ψ) of dipterocarp individuals in each forest type: a) Alluvial Forest, b) Sandstone Forest and c) Kerangas Forest.

Fig. S3. Relationship between tree height and diameter for dipterocarp trees of the studied species in the Alluvial Forest (blue), Sandstone Forest (orange) and Kerangas Forest (red).

Methods S1. Description of the water flow meter used for maximum hydraulic conductance measurements.

We used a custom thermal water flow meter in series with the sample to measure water flow (Miller & Small, 1982; Ashauer *et al.*, 1999). This method is the same as that used in Bronkhorst's Liqui-Flow sensors, which are also used in the Xylem Plus embolism meter (Bronkhorst S.A.S, France). The flow meter was calibrated against flow rates measured with a precision balance and these measurements were highly correlated (Pearson's r= 0.95). We measured pressure with a pressure sensor (26PCCFA6D, Honeywell). We controlled pressure in the hydraulic apparatus using a small 12V air pump (Rob10-398, SparkFun) to pressurise the air in contact with distilled water inside a container connected to the sample. We controlled the air pump, flow meter and pressure sensor within our hydraulic apparatus using a custom-built controller and data logger with an AVR-branded microcontroller (ATMEGA328P, Microchip).

Table S1. Tree density (individuals per hectare), species dominance (% basal area) and dominance rank (by basal area) within all species for each of the studied species in each Forest type. Total sampling area is 12 ha per forest type, with all trees with diameter at breast height more than 5cm measured. The habitat of each species is the habitat where the species has the highest dominance and is highlighted in bold. *S. multiflora and S. smithiana* have high dominance in two habitats. Total number of species in the Alluvial Forest is 489, in the Sandstone Forest 443 and in the Kerangas Forest 262. A dominance rank of 5 in the Alluvial Forest means that species has the 5th highest total basal area in that habitat.

	Tree density			Dominance			Dominance Rank		
Species	Alluvial	Sandstone	Kerangas	Alluvial	Sandstone	Kerangas	Alluvial	Sandstone	Kerangas
Cotylelobium melanoxylon	0.0	3.3	**82.8**	0.0	0.2	**8.7**	0.0	78	**2**
Dipterocarpus acutangulus	0.7	**47.3**	0.8	0.0	**8.7**	0.2	338	**2**	56
Dipterocarpus caudiferus	**12.8**	0.1	0.0	**3.6**	0.0	0.0	**5**	306	0.0
Dipterocarpus grandiflorus	0.0	**15.1**	2.6	0.0	**1.7**	0.2	0.0	**13**	63
Dipterocarpus kunstleri	**31**	0.1	0.2	**2.5**	0.0	0.0	**8**	207	257
Hopea beccariana	0.0	**34.2**	16.8	0.0	**3.4**	0.8	0.0	**5**	28
Parashorea tomentella	**26.2**	0.0	0.0	**14.2**	0.0	0.0	**1**	0.0	0.0
Shorea johorensis	**10.6**	0.0	0.0	**11.9**	0.0	0.0	**2**	0.0	0.0
Shorea macroptera	2.8	**21.9**	0.0	0.7	**1.3**	0.1	23	**14**	100
Shorea multiflora	0.0	**144.7**	**174.4**	0.0	**13.3**	**13.9**	0.0	**1**	**1**
Shorea smithiana	**3.0**	**5.8**	0.0	**2.1**	**2.0**	0.1	**11**	**10**	73
Shorea xanthophylla	**115.2**	0.0	0.0	**6.8**	0.0	0.0	**4**	0.0	0.0
Vatica micrantha	0.0	26.7	**37**	0.0	0.6	**1.4**	0.0	32	**16**

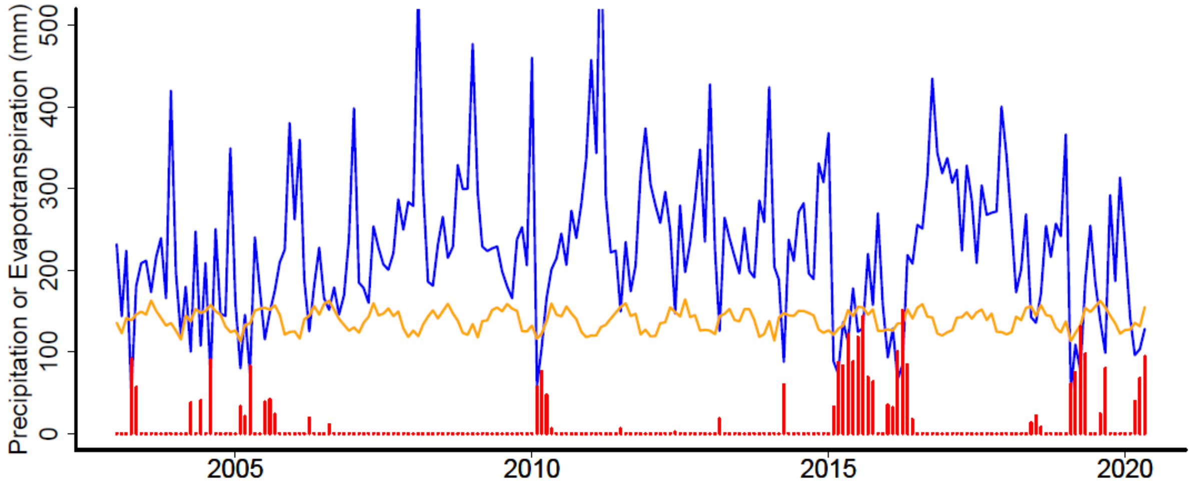

Figure S1. Time series of precipitation (blue line), evapotranspiration (orange line) and climatological water deficit (red line, also in mm; calculated as in (Barros *et al.*, 2019)) for the Sepilok region from 2003 to 2020. Note episodes of climatological water deficit are fairly uncommon and rarely exceed 100mm. Data is from "ERA5-Land monthly averaged data" product (Copernicus Climate Change Service, 2019).

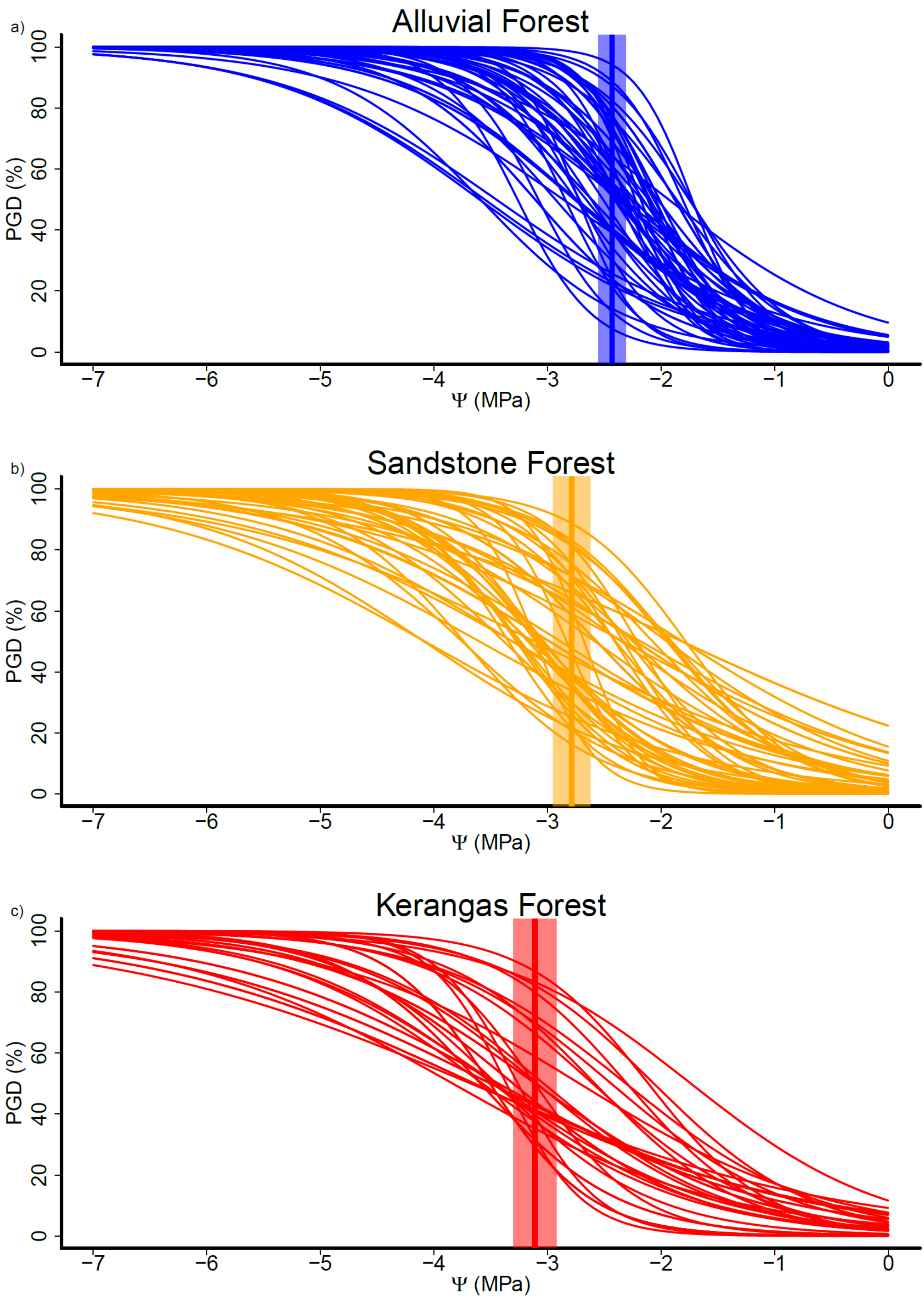

Fig. S2. Increase in embolism (PGD – percentage air discharge) with increasing branch xylem water potential (Ψ) of dipterocarp individuals in each forest type: a) Alluvial Forest, b) Sandstone Forest and c) Kerangas Forest. Each line represents one individual; vertical lines indicate the forest type

mean P50 (Ψ leading to 50% xylem embolism) and the shaded envelope marks the 95% confidence interval for mean P50.

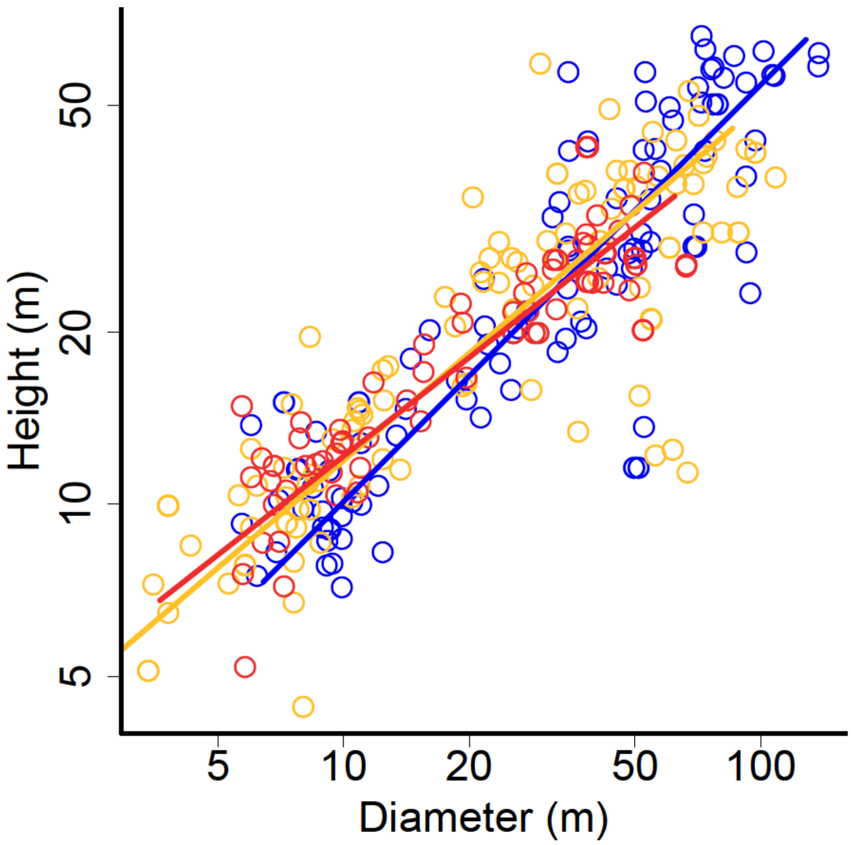

Fig. S3. Relationship between tree height and diameter for dipterocarp trees of the studied species in the Alluvial Forest (blue), Sandstone Forest (orange) and Kerangas Forest (red). Lines are the best fit models from standardized major axis regressions with forest type affecting the log transformed scaling of tree height and diameter. Scaling slopes ± CI for the alluvial, sandstone and kerangas forests are 0.73 ± 0.07, 0.62 ± 0.06 and 0.57 ± 0.06, respectively and the elevations are 0.27 ± 0.11, 0.45 ± 008 and 0.51 ± 0.08, respectively.

References

Ashauer M, Glosch H, Hedrich F, Hey N, Sandmaier H, Lang W. **1999**. Thermal flow sensor for liquids and gases based on combinations of two principles. *Sensors and Actuators A: Physical* **73**: 7–13.

Barros F de V, Bittencourt PRL, Brum M, Restrepo-Coupe N, Pereira L, Teodoro GS, Saleska SR, Borma LS, Christoffersen BO, Penha D, *et al.* 2019. Hydraulic traits explain differential responses of Amazonian forests to the 2015 El Niño-induced drought. *New Phytologist* **223**: 1253–1266.

Copernicus Climate Change Service. **2019**. ERA5-Land monthly averaged data from 2001 to present. URL: https://cds.climate.copernicus.eu/cdsapp#!/dataset/reanalysis-era5-land-monthly-means?tab=overview.

Miller TE, Small Hamish. **1982**. Thermal pulse time-of-flight liquid flow meter. *Analytical Chemistry* **54**: 907–910.
